# Spatial Human Development Index in China: Measurement and Interpretation Based on Bayesian Estimation

**DOI:** 10.3390/ijerph20010818

**Published:** 2023-01-01

**Authors:** Xiang Luo, Jingjing Qin, Qing Wan, Gui Jin

**Affiliations:** 1 College of Public Administration, Central China Normal University, Wuhan 430079, China; 2 School of Management, Wuhan Institute of Technology, Wuhan 430025, China; 3 School of Economics and Management, China University of Geosciences, Wuhan 430078, China

**Keywords:** HDI, Bayesian estimation, spatial spillover

## Abstract

The development of urban agglomerations dominated by the service industry is an important driving force for further sustainable economic growth of China. Spatial analysis marked by population density and regional integration is an essential perspective for studying the human development index (HDI) in China. Based on Bayesian estimation, this paper examines the influence of a spatial factor on HDI by using a spatial hierarchical factor model within the framework of Sen Capability Approach theory, overcoming the neglect of spatial factors and their equal weight in traditional measurement of HDI. On this basis, the HDI including the spatial factor was measured based on the panel data from 2000 to 2018. The results reveal that (1) provinces with high population densities and regional integration have higher rankings and low uncertainties of HDI, which can be attributed to the improvement of education weights; (2) HDI has a certain spatial spillover effect, and the spatial association increases year by year; (3) robust test by using nighttime lighting as an alternative indicator of GDP supports that the spatial correlation is positively related to HDI ranking. The policy recommendations of this paper are to remove the obstacles for cross-regional population mobility and adjust the direction and structure of public expenditure.

## 1. Introduction

It has been generally recognized by the government and academia that promotion of economic growth by urban agglomerations may be one of the most important forces that drive sustainable economic growth in the future. It is an objective rule that population and capital are increasingly concentrated in large cities and metropolitan areas with the development of urban agglomerations worldwide, which may be ascribed to the economic agglomeration and regional integration required by a service-dominated economic structure. In 2019, service industry accounted for 53.92% of the GDP in China, and its employment share also reached 47.40% of the total, which far exceeds the industrial employment share (27.50%) and the agricultural employment share (25.10%) (This indicator comes from *China Statistical Yearbook 2020*). It can be expected that in the structural transformation of China’s economy, the share of service industry will be further increased, and the unbalanced spatial distribution of population (human capital) will be further intensified. It is critical to determine how China’s regional development and spatial reshaping of population will affect the regional welfare levels. Evidently, answering this question will facilitate a better understanding on the pattern of China’s population flow and economic agglomeration, as well as more reasonable decision making on whether to provide reasonable policy guidelines for regional economic development.

Traditionally, the measurement of human development index (HDI) has been focused on some “visible” indicators such as longevity, education and income. In recent years, the role of spatial factors has aroused increasing attention in the measurement of HDI [1,2], which is mainly achieved by “amplifying” the externality of human capital through population density and reducing interregional coordination costs through regional integration (the externality of human capital means that the increase in a person’s educational level not only raises his own income level, but also generates the knowledge spillover effect in interaction with others, thus raising the overall income level). In fact, the research on HDI may be studies of spatial welfare in essence. The spatial welfare theory believes that the spatial pattern (total volume, density and distance), which is constituted by the initial material accumulation (social wealth and natural resources) and evolution of regional interaction, will ultimately determine the capacity for sustainable development of humans when basic security and livelihood are ensured. Particularly for a country as large as China, whose population is over 1.4 billion and land area is nearly 10 million square kilometers, it is very challenging to accurately evaluate the impact of large intra-national differences on welfare levels without taking spatial factors into account, which may result in inaccurate estimations (often overestimation) of HDI in certain regions. In addition, in terms of methodologies, the “priori” distribution in the Bayesian estimation provides a very efficient analytical tool including spatial factors for HDI measurement based on the available data. Based on previous studies, the core purpose of this paper is to reveal the impact of spatial factors such as population density and regional integration on regional welfare levels in China by constructing a spatial HDI, which is compared with the traditional HDI, and the reasons for the difference will be dissected as well.

With provincial panel data in China from 2000–2018, this paper establishes a spatial HDI using the spatial hierarchical factor model within the framework of Sen Capability Approach. We found that spatial HDI in provinces with high population densities and regional integration tends to have higher rankings than the traditional HDI once the key spatial factors such as population and geography are included in the HDI calculation process in the “prior” form of Bayesian estimation. In addition, population density and regional integration were found to help reduce the uncertainty in regional welfare improvement after the generation of confidence intervals for the spatial HDI through Markov Chain Monte Carlo (MCMC) sampling. Besides, the estimation results of the spatial autoregressive model (SAR) confirm the spatial spillover benefits of HDI. Finally, in the robustness test, we used the data of nighttime lighting as a proxy indicator for GDP to construct the spatial HDI, and the results are consistent with the previous ones except for slight variations in the ranking of several provinces, which again indicates that provinces with higher population density and regional integration have higher rankings. The findings of the present study are expected to help remove the barriers for cross-regional population movement and re-adjust the direction and structure of public expenditure.

The paper is structured as follows. Section 2 reviews the literature on spatial HDI and further explains the possible contributions of this paper. Section 3 introduces the measurement methods used and explains the data sources, and on this basis elaborates the differences between spatial HDI and traditional HDI. Section 4 presents the robust test by utilizing the data of nighttime lighting. Section 5 draws the main conclusions and policy recommendations.

## 2. Literature Review

For the measurement of welfare, HDI [3], which was proposed by the United Nations Development Programme (UNDP), has been widely used for its simplicity, which represents a very convenient analytical tool for assessing the level of welfare in a country or region. The proposal of HDI has brought remarkable policy implications for regional development (including national development). For example, the Irish government took HDI as a criterion and provided more assistance to countries classified as “low human development” according to HDI [4]. At the corporate level, Merck and Co. Inc. sold low-cost drugs to almost all countries classified as “low human development” at much lower prices than for other countries [5]. Recent empirical evidence from China suggests that China’s achievements in HDI are not only due to economic growth but are also ascribable to the particular attention that Chinese government has paid to the investment in education and health care for poverty reduction compared with other developing countries [6]. However, China has not invested enough in the central and western regions, which may be the reason for the slow improvement of HDI in China [7] (in this study, China’s HDI was 0.763 in 2017, making it one of the “high human development” countries. However, China’s education score was only 0.658, and is even lower than the world average score of 0.661, which is mainly attributed to the low scores in central and western regions of China).

However, the traditional HDI is focused on “visible” indicators such as longevity, education and income [8]. Therefore, the aggregation of China’s population in a few cities or urban circles may leave a false impression that excessive population aggregation is detrimental to regional welfare, causing problems such as traffic congestion, inadequate housing and environmental pollution. In fact, the HDI of China has undergone continuous rising since the reform and opening up. China evolved from a very “low human development level” to a “high human development level” and reached up to 0.763 in 2017, making China one of the “high human development” countries; besides, the HDI of all provinces in China has generally increased [9,10]. The above phenomena reveal that although the HDI of China is increasing in general, it tends to show gradual differentiation between regions, the extent of which may be related to spatial factors that are “rightfully” neglected by the traditional HDI. Some insights have been provided by the past literature. For example, Hicks first added some indicators, such as poverty incidence and the Gini coefficient, which can reflect the poverty dimension and equity dimension to the traditional HDI [11]. Additionally, Zhou followed his improved method; based on provincial panel data, he proposed that although China’s overall HDI shows an upward trend, there are significant differences among regions, and the eastern regions with better environment have obviously better performance in poverty alleviation or equity than the central and western regions [12]. A similar study was carried out by Li; GIS and statistical methods such as coefficient of variation, Moran’s I and spatial regression are used in this study [13]. The results illustrate that the overall disparity in HDI declined, but the spatial concentration increased.

In addition to economic and social factors, regional differences in China are also manifested in the natural environment; a growing number of studies integrate HDI and sustainability issues [14]. Therefore, some other literature investigated the regional HDI of China from the perspective of the natural environment [15,16]. For instance, Chen et al. introduced a per capita CO_2_ emission indicator into HDI as the fourth dimension of equal weight (like Vega and Urrutia [17]) to study the Beijing-Tianjin-Hebei region and assessed the sustainability dynamics of the BTH urban agglomeration at the city scale from 2000 to 2015 [18]. The results showed that the HSDI of the BTH urban agglomeration increased and the overall sustainability improved; economic and social sustainability had an increasing trend, but environmental sustainability exhibited a downward tendency. In a further study of Li and Wang, they constructed an ecological input index (EII) that comprises resource consumption and pollution emissions and incorporated it into HDI [19]. Based on the provincial data, spatial exploration and spatial econometric analysis revealed that the Moran’s I of China’s HDI is significantly positive, and there is no *α*-convergence in the regional HDI, but there are significant absolute β-convergence and conditional *β*-convergence. Furthermore, some studies also combined the ecological footprint and the human development index [20], and the results of this study show that there is great potential for improving the efficiency of sustainable development in China, and China’s regions presented different upward trends in the order of the western, central, and eastern regions, from high to low [21]. Conclusions generally consistent with previous ones have been proposed: China is still at a stage of “high consumption and low welfare” development, and HDI is lower in regions with higher consumption of natural resources.

Since almost all relevant literature has reached the consistent conclusion that there are significant spatial variations in HDI of China, it is critical to elucidate how to incorporate spatial factors into the measurement of HDI and what may be the similarities and differences between measurement of HDI with and without the consideration of spatial factors. It is a pity that the existing discussion on China’s HDI is mostly focused more on results. Whether it is comparison of HDI at the regional level or the division of it at a geographical scale (such as Gini or Taylor coefficient), spatial factors are unexceptionally exogenized in existing empirical studies. Another reason for this deficiency in literature may be ascribed to the principle of equal weighting of indicators in traditional HDI setting [22]. In fact, once spatial factors are taken into account, calculation of HDI with the equal weighting principle will be obviously unreasonable, since the development level of a region is not only determined by the local economy, education and health, but it is also closely associated with the development of surrounding areas (degree of integration). For this problem, some studies attempted to put weight to HDI through principal component analysis (PCA) [23]. However, this approach also has distinct drawbacks. The core approach of PCA is to assign weight by dimension reduction. However, dimension reduction is not so important in the setting of HDI because it usually involves not so many basic indicators, and even if more basic indicators are utilized due to research need, it may bring the problem of high collinearity, and it also violates the principle of concision and intuitiveness during the setting of HDI [24].

In summary, based on previous studies, this paper mainly aims to reveal the impact of population density and regional integration on China’s HDI by constructing a HDI including spatial factors and compare the effect with that of traditional HDI as well as explain the differences. Accordingly, the possible innovations and contributions of this paper are as follows. First, a spatial hierarchical factor analysis model is constructed on the basis of Bayesian estimation, which incorporates spatial heterogeneity (population and geography) into HDI measurement in the form of a priori assumption. The HDI constructed in this paper not only directly reflects the influence of spatial factors (particularly population density and regional integration) on regional welfare, but also overcomes the drawbacks of the traditional HDI with equal weighting. Second, spatial factors are incorporated in the measurement of China’s HDI, which is compared with the measurement of traditional HDI. Besides, this paper provides detailed explanations on the similarities and differences between these two methods and evidence for the spatial heterogeneity of HDI in China. The findings may enrich the understanding of human development levels in different regions in China as well as facilitate the setting of more rational population policies and regional development policies in the future.

## 3. Materials and Methods

### 3.1. HDI Constructed in the Spatial Hierarchical Factor Model

Following the concept of Høyland et al. [1], the specific form of the basic hierarchical factor analysis model is given as

(1) $$Y_{ij}=\mu_{j}+\lambda_{j}\delta_{i}+\varepsilon_{ij}$$

In Equation (1), the subscript *j* represents the observable variable and *i* represents the region (j=1,2,3,4, i=1,2,⋯,31); on the left side of the equation, $Y_{ij}$ is the observed variable (based on the idea of Hogan and Tchernis [25], to stabilize the variances, $Y_{ij}$ is obtained from the HDI square root transformation. Of these, the HDI indicators are already in “per capita” form), whereas on the right side, $\delta_{i}$ indicates the potential HDI of a certain region, which is also the core measure value of the model; $\mu_{j}$ is the sample mean of variable *j*; $\lambda_{j}$ is the factor loading, which is specifically the covariance between the human development level $\delta_{i}$ and the observable variable $Y_{ij}$; $\varepsilon_{ij}\sim N(0,\sigma_{j}^{2})$ is a perturbation term obeying the normal distribution and satisfying the independent identically distributed (iid) assumption, meaning that the human development level $\delta_{i}$ is only related to the observable variable $Y_{ij}$.

On the basis of Equation (1), spatial factors could be incorporated to construct a spatial hierarchical factor model. First, the conditional distribution of HDI with the inclusion of spatial factors is given as

(2) $$\delta_{i}|\delta_{j}\sim N(\sum_{j\in \phi_{i}}\omega \delta_{j},\upsilon )$$

In Equation (2), ω is the degree of regional integration, $j\in \phi_{i}$ is defined as the domain collection of region *i* and υ is the conditional variance. By normalizing the conditional variance as υ=1 and assuming that regional integration is accepted into this model by the *queen’s law* of “bordering or not bordering”, the marginal distribution of Equation (2) is obtained

(3) $$\delta \sim N(0,{\left(I-\omega W\right)}^{-1})$$

*W* in Equation (3) is a non-random spatial weight matrix of order *N*×*N*, $W=\sum_{j=1}^{N}w_{ij}=\left[\begin{array}{cccc} w_{11} & w_{12} & \cdots & w_{1N}\\ \cdots & \cdots & \cdots & \cdots \\ w_{n1} & w_{n2} & \cdots & w_{nN} \end{array}\right]$. *w_ij_* = 1 refers to the bordering of two regions and *w_ij_* = 0 refers to the contrary situation. Next, the regional population density *m_i_* is introduced into Equation (3), because it is a very important variable affecting the HDI in existing literature [2]. On the one hand, regional population density directly reflects the degree of economic agglomeration (including the externality of human capital). On the other hand, areas with high population densities also face relatively less uncertainty in welfare changes. Ultimately, the vector form of HDI constructed by means of the spatial hierarchical factor model can be expressed as (for the convenience of statement, the spatial HDI in the following part of this paper refers to the HDI constructed by the spatial hierarchical factor model)

(4) $$\{\begin{array}{c} Y|\delta \sim N(\mu +\Lambda \delta ,M^{-1}⊗\Sigma )\\ \delta \sim N(0,M^{-\frac{1}{2}}\psi M^{-\frac{1}{2}}) \end{array}$$

In Equation (4), Y is the superimposed vector of $Y_{ij}$ on the direction of *j*; $\Lambda =I_{N}⊗\lambda$, where *I_N_* is a unit matrix of the order *N*×*N*; Σ is a diagonal matrix with $\delta_{j}^{2}$ as the element on the main diagonal; $\psi ={\left(I-\omega W\right)}^{-1}$; *M* is a matrix of the order *N*×*N* of population in a certain region, with the elements of (*m*_1_*, m*_2_, …, *m_N_*) on the diagonal.

### 3.2. MCMC Sampling

Based on Equation (4), we referred to the MCMC sampling used by Hogan and Tchernis to calculate the HDI for all regions [25]. The specific steps are as follows (the estimation of all parameters can be found in the appendix “POSTERIOR SAMPLING ALGORITHMS” of Hogan and Tchernis [25]).

Step 1: Estimation of the factor loading *λ*. 1*_N_* is supposed to be a *N*×1 vector with all elements equal to 1. For any *λ_j_*, the estimation equation is $Y_{j}-{1_{N}}^{\prime }\mu_{j}=\lambda_{j}\delta +\varepsilon_{j}$, where $Y_{j}$ is an *N*×1 vector of the observable variable $Y_{ij}$ and $\varepsilon_{j}\sim N(0,\sigma_{j}^{2}/M)$. The priori distribution is required to be $\lambda_{j}\sim N(a,A)$, in which *a* = 0 and *A* = 4000. Therefore, the posterior distribution of $\Lambda_{j}$ can be derived from the conditional distribution *N* (*b*, *B*) in Equation (5).

(5) $$\{\begin{array}{c} B={\left(1/A+\delta^{\prime }M\delta /\sigma_{j}^{2}\right)}^{-1}\\ b=B[a/A+\delta^{\prime }M(Y_{j}-1_{N}\mu_{j})/\sigma_{j}^{2}] \end{array}$$

Step 2: Estimation of the potential human development *δ.* The estimation equation is supposed to be $Y-\mu ⊗1_{N}=\Lambda \delta +\varepsilon$, where *Y* is an *N_J_* ×1 vector of the observable variable *Y_i_* and $\varepsilon \sim N(0,M^{-1}⊗\sum )$. It can be deduced from Equation (4) that the priori distribution of *δ* is $\delta \sim N(0,M^{-\frac{1}{2}}\psi M^{-\frac{1}{2}})$. Therefore, the posterior distribution of δ can be derived from the conditional distribution *N* (*d*, *D*), as shown in Equation (6).

(6) $$\{\begin{array}{c} D={\left[{\left(M^{-\frac{1}{2}}\psi M^{-\frac{1}{2}}\right)}^{-1}+\Lambda^{\prime }{\left(M^{-1}⊗\sum \right)}^{-1}\Lambda \right]}^{-1}\\ d=D[\Lambda^{\prime }{\left(M^{-1}⊗\sum \right)}^{-1}(Y-\mu ⊗1_{N})] \end{array}$$

Step 3: Sampling of the spatial correlation *ω* with the Metropolis-Hasting algorithm in MCMC. The posterior distribution of *ω* is assumed to be $\pi (\omega )=N(0,1000)I(\eta_{1}^{-1}<\omega <\eta_{N}^{-1})$. $\eta_{1}^{-1}$ and $\eta_{N}^{-1}$ represent the minimum and maximum eigenvalues of the spatial weight matrix *W*, respectively. Therefore, the density function of *ω* can be expressed as $\eta_{N}f(\delta |\psi (\omega ))\pi (\omega )$. Furthermore, the proposal density is assumed as $q(\omega^{\prime }|\omega )\sim N(\omega ,\rho^{2})$. Then, $\omega^{\prime }$ can be achieved from a random walk model $\omega^{\prime }=\omega +\varepsilon$, in which ε is a perturbation term complying with $\varepsilon \sim N(0,\rho^{2})$ and $\rho^{2}$ is a tunable parameter. As a result, the *ω* value range generated by the sampling is limited to $\eta_{1}^{-1}<\omega <\eta_{N}^{-1}$. Accordingly, the possibility of being accepted for $\omega^{\prime }$ is

(7) $$\min\left\{1,\frac{f(\delta |\psi (\omega^{\prime }))\pi (\omega^{\prime })q(\omega |\omega^{\prime })}{f(\delta |\psi (\omega ))\pi (\omega )q(\omega^{\prime }|\omega )}\right\}$$

### 3.3. Data Sources and Explanation for China’s Spatial HDI

Before measuring and explaining China’s spatial HDI based on the method proposed in this paper, we first review the traditional HDI. In terms of theoretical origins, the breakthrough in welfare research should be attributed to the pioneering paper of Sen [26]. Starting from his research, a feasibility capability theory has been gradually developed in economics to measure welfare, the core opinion of which is that welfare should be considered as the human ability to freely strive for and expand a worthwhile life. Later, the United Nations Development Programme (UNDP) proposed the concept of the Human Development Index (HDI) in 1990, which marks the beginning of applied research on the measurement of human welfare within the framework of feasibility capability theory. In contrast to the unidimensional GDP, HDI holds that development is not only a result of economic data but also the “visible well-being of people”. Guided by this concept, three crucial indicators (life expectancy, education degree and income level) are taken to determine the development degree, which have been widely used in studies that estimated the welfare level and ranking of countries or regions [27,28].

Specifically, in the HDI proposed by UNDP, three secondary indicators are constructed with four important variables: Life Expectancy Index (*LEI*), Education Index (*EI*) and Income Index (*II*). Then, a complete HDI can be constructed with the geometric average (equal weighting) as $HDI=\sqrt[{3}]{LEI\times EI\times II}$. These three secondary indicators are as follows.

(8) $$LEI=\frac{LE-20}{85-20}$$

(9) $$EI=\frac{MYSI/15+EYSI/18}{2}$$

(10) $$I=\frac{\ln(GNIpc)-\ln(100)}{\ln(75000)-\ln(100)}$$

In these equations, *LE* refers to the life expectancy at birth; *MYS* is the mean years of schooling; *EYS* stands for the expected years of schooling; *GNIpc* is the gross national income per capita after the adjustment of purchasing power. The following are the detailed data sources for this study. (1) *LE* data. Since the *LE* data could only be available in 2000 and 2010 censuses and the annual 1% population sample survey in China, the *LE* data for other years are properly substituted by the data of average life expectancy of adjacent years. As a result, the *LE* data of 2001–2002, 2003–2005, 2006–2009, 2010–2012, 2013–2015, 2016–2018 are replaced by the average life expectancy data calculated in 2000 or published in the *China Human Development Report* in 2005, 2013 and 2016, respectively. (2) *MYS* data. According to the results of the national population census and *China Statistical Yearbook*, the proportions of the population with various educational levels, including primary school, junior middle school, senior high school, secondary specialized school and college or above, can be obtained at the national or local level. The schooling years of various levels are 6, 9, 12 and 16; the *MYS* can be calculated at the local level in the census year, which can then be calibrated with the national *MYS* to obtain a relatively accurate *MYS* at the local level. (3) *EYS* data. Since there is a high correlation between expected education level and average education level, the education data at the local level can be obtained from the data at the national level assuming that the proportion between the average years of schooling and *EYS* is consistent with the national level. (4) *GNIpc* data (the education index and income index are calculated with reference to *China Human Development Report* (*2019*). (https://www.cn.undp.org/content/china/zh/home/library/human_development/national-human-development-report-special-edition.html) (accessed on 1 October 2021). The data of per capita GDP in various regions can be obtained according to the *China Statistical Yearbook* for each year, which was measured by the RMB price of that year. Based on national-level data (from the UNDP website), the proportional relationship between per capita GDP measured at current-year RMB prices and GNP measured at current-year PPP prices can be deduced, which is equivalent to the exchange rate conversion factor. Based on this conversion coefficient, the GNP data measured with current-year PPP prices for each region can be obtained.

It should be noted that two sets of physical geographic data are applied in this paper besides the above-mentioned economic and social development data. (1) Nighttime lighting data are derived from two sets of remote sensing data: DMSP-OLS remote data of nighttime lighting (1992–2013) and NPP-VIIRS remote data of nighttime lighting (2012–2018). The DMSP-OLS data record the brightness of nighttime lighting from 8:30 p.m. to 10:00 p.m. in each region from 1992 to 2013 and exclude natural firelight, transient light and other background noises to ensure that the recorded data can represent the brightness of artificial lighting. The values of the lighting brightness range from 0 to 63. A higher value indicates higher brightness, which is interpreted as more prosperous economic activities in this region. NPP-VIIRS collects the radiation images of the Earth’s land, atmosphere and oceans in visible and infrared bands from 2012 to 2018 with the higher spatial resolution (500 m) for a more accurate description of the distribution of small-scale illuminations on earth surface. Therefore, the DMSP-OLS and NPP-VIIRS remote sensing data of nighttime lighting are inter-calibrated in order to obtain a remote sensing dataset of nighttime lighting of a longer time span. Both sets of remote sensing data of nighttime lighting are derived from the National Oceanic and Atmospheric Administration (NOAA) website (https://www.ngdc.noaa.gov/eog/ (accessed on October 1, 2021)). (2) Spatial Weight Matrix. The geographic factors (such as proximity and inverse distance) in the spatial weight matrix are generated with the Generate Spatial Weights tool in the Spatial Statistics Module in the ArcGIS 10.2 software toolbox and extracted by MATLAB (R2020b) to obtain a 31 × 31 non-random spatial weight matrix. 

## 4. Results

### 4.1. Spatial HDI Ranking vs. Traditional HDI Ranking

**Figure 1:**
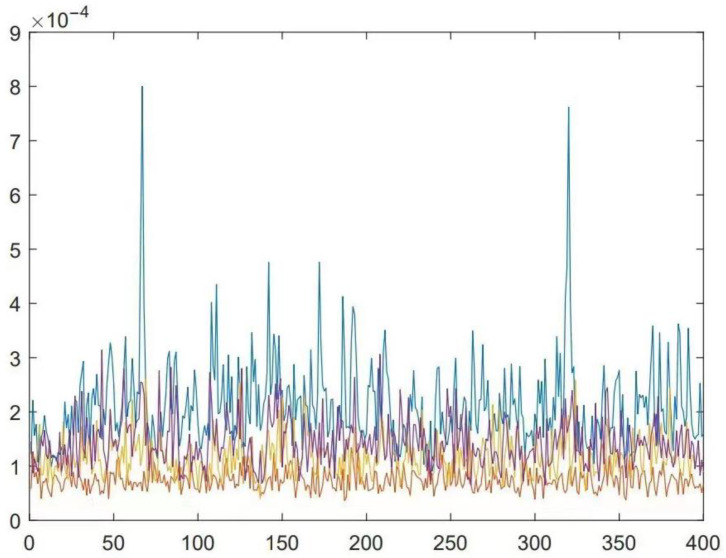
Estimation process of the parameter $\sigma^{2}$.

**Figure 2:**
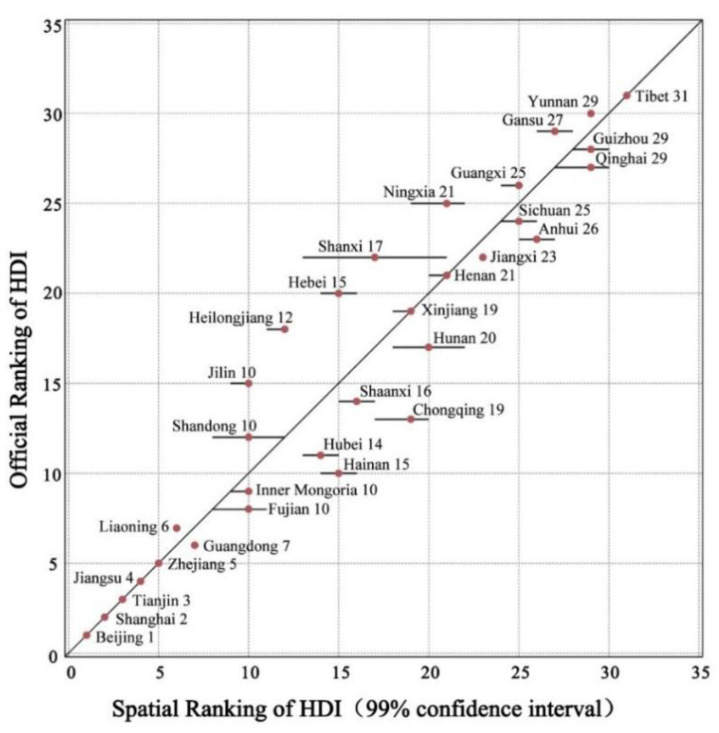
Comparison of Spatial HDI and Traditional HDI (mean values from 2000 to 2018).

After the initial convergence stage of 50 iterations, we conduct 400 iterations and implement the ranking by means of the posterior distribution of factor scores, so that the samples can be achieved from the posterior distribution of ranking of provinces and cities in China. Figure 1 shows the estimation process of parameters. Figure 2 and Table 1 manifest the difference between spatial HDI and traditional HDI.

In Figure 2, the dotted grid divides ranks of 0–5 (the first rank), 6–10 (the second rank) and etc.; the solid dot represents the corresponding position of a specific province in two HDIs. In addition, the horizontal line passing through each solid dot represents the 99% confidence interval of HDI for a specific province; the 45-degree line from the origin means that the spatial HDI has the same raking with the traditional HDI. It can be seen from Figure 2 that although the ranking of spatial HDI is uncertain, the confidence intervals of most provinces are within the same ranking level, whereas only a few provinces have confidence intervals even spanning three ranking levels. For example, the 99% confidence interval of spatial HDI in Shanxi is (13, 21), but it ranks below the median in two HDIs with different computing methods, which means that the spatial HDI used by us does not essentially change the ranking of the traditional HDI (for example, the provinces with lower scores rank higher by spanning several levels). Furthermore, we also observe that regions with high regional integration degree reveal two HDIs which are generally consistent, such as Shanghai, Beijing and Guangdong, respectively located in the Yangtze River Delta, Beijing-Tianjin-Hebei region and Guangdong-Hong Kong-Macao Bay Area where the regional integration plays a central role. Therefore, these provinces are located closer to or at the 45° line with lower uncertainties (the rankings of Tibet in both HDIs are also consistent, mainly due to its unique geographical location and economic situation, rather than spatial correlation. Therefore, in this paper, Tibet can be treated as an “outlier”). What’s more, it needs to be pointed out that, compared with the traditional HDI (ranking 20), the 99% confidence interval of the spatial HDI in Hebei ranks above the median (14, 16). In addition to the impact of higher education year, this may also be closely related to the enhancement of regional radiation effect under the coordinated development strategy of Beijing-Tianjin-Hebei integration.

Table 1 lists the 10 provinces with the largest gaps between the spatial HDI ranking and traditional HDI ranking. The difference in the HDI value between them may be attributed to the fact that this paper does not calculate HDI by equal weighting; instead, it assigns the “space” with a higher weight. Specifically, among the traditional HDI, the health index, education index and income index account, three secondary indicators, account for one third of weight, respectively. Among the spatial HDI, the weight of the education index has relatively increased to 0.3544. In the ranking of HDI, it can be found that compared with the traditional HDI, provinces with a higher education level have a higher ranking in spatial HDI. Taking Shanxi as an example, its traditional HDI ranks 22, whereas it’s ranking is 17 in spatial HDI, which is up five places. According to the data of the Seventh Population Census (hereinafter referred to the Seventh Census), the average educational year for the population aged 15 and over in Shanxi is 10.45, ranking fourth in China (only next to Beijing, Shanghai and Tianjin). A similar situation also occurs in Jilin, Heilongjiang, Hebei and other provinces. According to the traditional thinking, the ranking of provinces with low economic growth in HDI are not believed to increase rapidly. However, the fact is that Northeast China has very rich human capital, which may significantly affect its ranking of spatial HDI. The Seventh Census shows that 16.75 percent of the population in three northeastern provinces have an associate college education or above, and the average educational year of the population over 15 years old is 10.16 years. The above two important indicators reflecting the education level of the population in this region are higher than the national average. Moreover, Northeast China is also one of regions with the highest urbanization rate (67.71%) in China and has good urban infrastructure, all of which is conducive to the improvement of its spatial HDI.

Correspondingly, the provinces with net population outflows have declined in the ranking of spatial HDI. In Table 1, Anhui, Hunan and Hubei rank 23, 17 and 11, respectively, in the traditional HDI, whereas their rankings of spatial HDI are 26, 20 and 14, all of which decline by three places. The common feature of the above three provinces is the large-scale population outflow with a low regional integration degree. According to the survey of the Seventh Census (2020), compared with 2010, the net population outflows of Anhui, Hunan and Hubei are 10.1685 million, 6.7504 million and 4.0258 million, respectively, making them the provinces with the largest population outflows in China. It is worth mentioning that Henan, with a net population outflow of 15.4913 million, is the largest province in China, but its rankings in two HDIs are relatively consistent, which may be related to the enhanced regional integration of Zhongyuan Urban Agglomeration in recent years. In addition, Henan also has relatively high urban population density. The relationship between urban population density and welfare improvement has been confirmed by existing research [29], which can offset the adverse impact of population outflow on HDI to some extent (based on the China Statistical Yearbook (2021), the urban population density of Henan Province in 2020 was 4994 people/km^2^, making it one of the relatively high urban population densities in China (ranked second)). Similarly, Chongqing and Sichuan in the Chengdu-Chongqing metropolitan area have urban population density of 2070 and 3158 people per square kilometer, respectively, which reveal the HDI of Chongqing is relatively overestimated. Although Sichuan is one of provinces with a large net outflow of population (7.3201 million), its population factor shows an obvious “center and periphery” relationship, which means that the provincial capital Chengdu has a strong siphon effect on peripheral cities and further influences the HDI in Sichuan. However, compared with the traditional HDI, the spatial HDI in Chongqing has declined by six rankings, which may be determined by spatial correlation. The population of long-term residents in Chongqing has not increased in the past 10 years, but it has a net outflow of 2.1087 million people, which reflects that the attraction of the Chongqing metropolitan area to the peripheral population and the retention ability of local population are relatively weak, and this negative spillover effect influences its ranking to a certain extent.

Generally speaking, all provinces with higher rankings in spatial HDI measured by our methodology exhibit two characteristics: high population densities (especially urban population density) and high regional integration levels. In this regard, our explanations are as follows. First, the population density reflects the positive effect of the externality of human capital on welfare. Theoretically, areas with the higher population densities have more employment opportunities, where complementarity can be established between different industries and types of employment (such as high and low skills), contributing to increases in real income.

**Figure 3:**
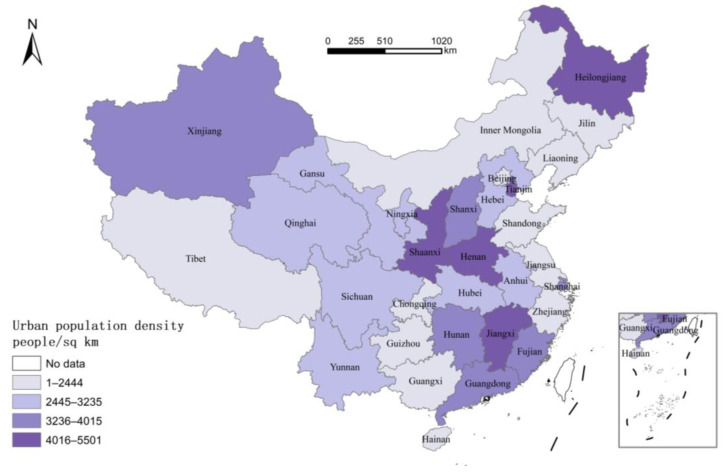
Urban Population Density by Province in 2020.

**Figure 4:**
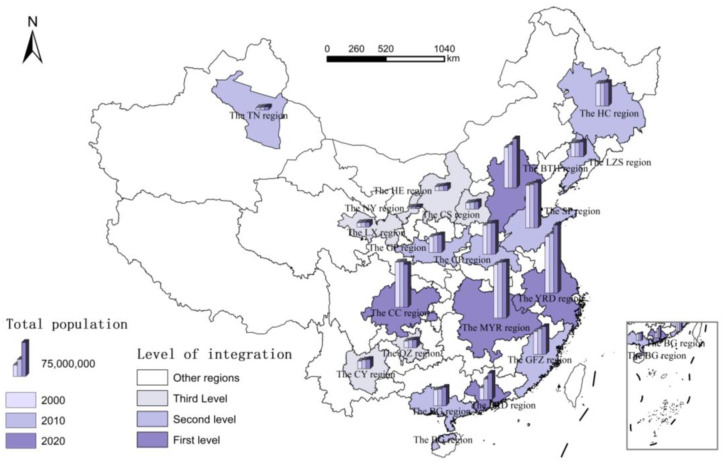
Regional Integration and Total Population (referring to *the Outline of the 14th Five-Year Plan and 2035 Vision for National Economic and Social Development of the People’s Republic of China*, the 19 national-level urban agglomerations (acronyms) in China are used to characterise the current state of regional integration, including five for optimisation and upgrading, five for development and growth, and nine for cultivation and development).

In addition, a high population density means good public service (Figure 3). Particularly, the investment in public resources, such as education and health care, not only directly improves the current welfare, but this also has important impacts on the human capital accumulation and life expectancy, which can “amplify” the externality of human capital in the long run. Second, the regional integration degree reflects the positive impact of resource allocation efficiency on welfare (Figure 4). In China, administrative interventions between regions for the sake of “competing for promotion” tend to cause distortions of resource allocation (such as capital bias and financial frictions). One benefit of the regional integration is that it largely reduces the intervention of administrative forces in the market and promotes the free movement of factors (at least within the region). On the other hand, it can also decrease the cost of addressing the externality between regions (such as pollution). Empirical studies have revealed that strengthening the management of regional integration by means of spatial regulation can promote social welfare to a large extent [30].

### 4.2. “Spatial Spillover” Effect Test

As a matter of fact, if traditional HDI is considered as the measurement of “current” welfare, the spatial HDI proposed in this paper portrays a picture of “future” welfare. In particular, with the advancement of China’s urbanization and marketization, it can be expected that not only will the flow of factors become freer, but also urban agglomeration will play a more remarkable role in driving regional economic development. Therefore, the spatial HDI proposed in this study, which includes both demographic and spatial factors, may more accurately portray the level of human welfare development in China. Further, in order to account for the significant influence of spatial factors on HDI, drawing on relevant research [31,32,33,34], a spatial autoregressive (SAR) model is established to test the existence of a “spatial spillover” effect in HDI as follows.

(11) $$HDI_{it}=\beta_{0}+\rho WHDI_{it}+\alpha_{i}+\alpha_{i}t+\varepsilon_{it}$$

In Equation (11), *WHDI_it_* is the spatial lag term of HDI for region *i*; *W* is a spatial weighting matrix as shown in Equation (3), and the coefficient *ρ* characterizes the spatial spillover effect of the HDI among neighboring regions on that of the tested region, which is the core mechanism to be verified in this paper; the subscript *i* (*i* = 1, 2,…, 31) represents the region, and *t* represents the time (*t* = 2000, 2010,…, 2018); *α_i_* is an individual effect; *α_i_t* is a time effect; *ε_it_* is a random disturbance term obeying the independent identical distribution. It should be noted that the HDI here is not what we have calculated in chapter one with the spatial hierarchical factor model. The reasons are as follows. First, in the spatial hierarchical factor model, we have already used the spatial weighting matrix once, whereas in Equation (11) there is another spatial weight matrix, which may lead to biased estimation results if the spatial weight matrix is used repeatedly on both sides of the econometric model. Second, the HDI calculated through the spatial hierarchical factor model includes confidence intervals, which may generate potential endogeneity due to measurement errors if regressed as explained variables. Third, considering that the purpose of Equation (11) is to verify the spatial spillover effect of HDI, the application of traditional HDI can also test the core mechanism.

**Figure 5:**
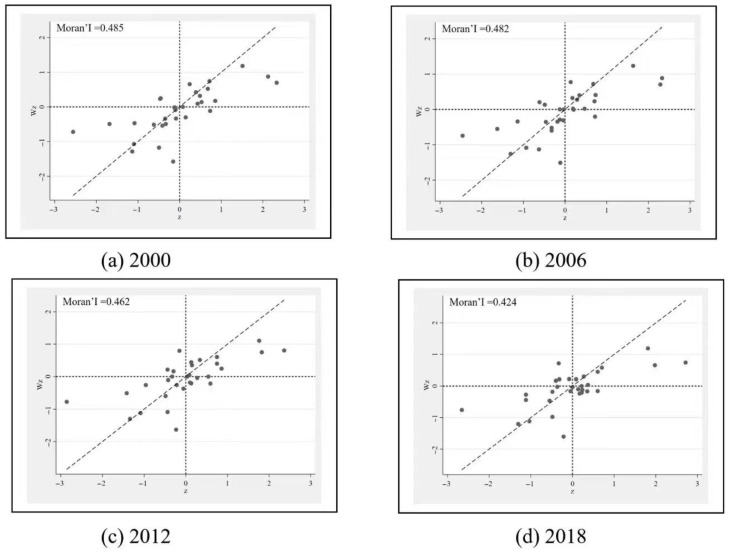
Scatter plot of Moran’s I in HDI.

Before the specific regression, we should firstly test the spatial correlation of the dependent variable HDI. The Moran’s I in HDI is set as $I=\frac{\sum_{i=1}^{31}\sum_{j=1}^{31}w_{ij}(HDI_{i}-\bar{HDI})(HDI_{j}-\bar{HDI)}}{S^{2}\sum_{i=1}^{31}\sum_{j=1}^{31}(HDI_{i}-{\bar{HDI)}}^{2}}$. *S*^2^ is the sample variance and *w_ij_* is the (*i,j*) element of the spatial weighting matrix in Equation (11). Figure 5 is a scatter plot of the Moran’s I in HDI for 2000, 2006, 2012 and 2018. As shown clearly in Figure 5, the Moran’s I in HDI is significantly greater than zero in all years, and the spatial autocorrelation test also strongly rejects the original hypothesis of “no spatial autocorrelation”.

From the estimation results in Table 2, both the fixed effect estimation of SAR and the GMM-SAR estimation with the exclusion of endogeneity reveal that there is a “spatial spillover” effect of HDI between regions; that is, changes in local welfare levels are affected by the neighboring regions, and the direction of this effect is consistent. This result can be explained from two aspects: namely, the bandwagon effect of economic growth, and the demonstration effect of public services. In China, economic growth is taken as a crucial indicator for assessing the performance of officials. Hence, local officials often have a strong incentive to develop the local economy. Besides, such an incentive is largely driven by their competitors, which is often referred to as the “Promotion Tournament” model. In this model, the activity of “competing for growth” between local governments undoubtedly will raise the income level and thus have a race-to-top impact on HDI. However, the spatial spillover effect of HDI caused by the “Promotion Tournament” model is likely to be short-term. In the long run, the HDI still tends to be determined by the public service level. In theory, if the local public services of a region are inferior to those of neighboring areas, the labor force will flow to areas with better public services, leading to losses of local economy and tax revenues. Accordingly, the local response is to improve the quality of public services. Obviously, the mechanism of “voting with feet” also ensures the race-to-top of HDI in the long run.

Furthermore, in order to capture the specific impact of spatial correlation between regions on HDI, we provide the cross-section regression results of SAR in Table 3. It can be found in the results that the estimate coefficient of spatial autocorrelation for HDI is increasing year by year, with a coefficient of 0.018 in 2000 and 0.176 in 2018, showing a 877.8% increase. It can be expected that the efficiency of cross-regional allocation of factors will be further improved in the process of urbanization and marketization in China. Accordingly, spatial factors will have more important impacts on the HDI.

### 4.3. Robust Test

In this section, we will construct the HDI by taking the nighttime lighting data as an alternative for GDP and perform the robustness test. The use of nighttime lighting data has the following advantages. First, the nighttime lighting data can more thoroughly reflect the spatial welfare of economic activities from the spatial dimension. Traditional economic statistical data with GDP as the core are generally based on administrative divisions, whereas the nighttime lighting data can effectively extract the spatial range of economic activities and be perfectly applied to assess the quality of regional economic development through the luminous intensity. Particularly for the service-dominated economy, the nighttime lighting data can record the information difficult to be captured in GDP, such as the informal economy and political preference, providing more real data about human activities (the nighttime lighting data were mainly obtained by the technological means and less depending on institutions and human resources. For example, Hodler and Raschky examined the impact of leaders’ local preferences on regional economic development based on the global panel data of nighttime lighting from 1992 to 2009 [35]. Their study revealed that the nighttime lighting intensity of a political leader’s birthplace was significantly enhanced during his inauguration and reign, whereas it fell back remarkably after he resigned). Second, the quality of nighttime lighting data is higher from the time dimension. A great challenge for utilizing GDP to record human economic activities is to ensure the consistency and comparability of data. As a matter of fact, the statistical framework of GDP is still in the process of constant revision. Especially for a large country like China, where the proportion and categories of its service industry are increasing, the adjustment of GDP accounting at least involves the changes in the sources of information, calculation methods, accounting scope and classification, which will undoubtedly affect the quality of data in the end.

**Figure 6:**
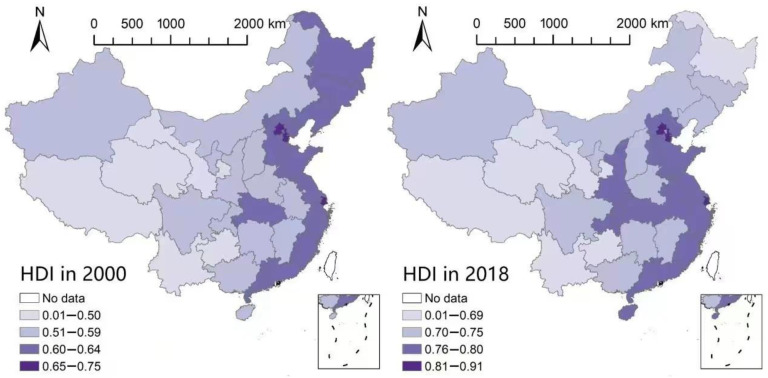
Spatial Distribution of HDI Constructed with Lighting Data.

Figure 6 reveals the spatial distribution of HDI constructed with the nighttime lighting data in 2000 and 2018. As can be seen from the figure, in the provinces with high population densities and good spatial location, the HDI is significantly improved compared with that in 2000. In addition, the HDI grows much faster in provinces located on the eastern coast and important economic zones or city agglomerations than in other provinces. On the contrary, the HDI decreases in cities with high population outflow and resource-based characteristics. All these results are in accordance with our findings about the spatial HDI in the previous sections. The robustness test results in Figure 6 reaffirm the fact that population size and spatial environment play essential roles in improving China’s overall welfare level as China’s economic development becomes increasingly dependent on the service industry and urban agglomerations.

## 5. Discussion

Although this paper has conducted some explanatory work on measuring the impact of spatial factors on HDI, some implicit policy recommendations can still be proposed. The experience of Chinese and global economic development indicates that the aggregation of the population in a few regions can promote regional integration [36]. According to the results of the seventh census, the migrant population in China is up to 376 million, among which 125 million people migrate across provinces, accounting for about one-third of the total migrant population. In addition, our results further demonstrate that population density and regional integration level have positive effects on the improvement of HDI, implying that the direction of policy adjustment should be conformed to the law of the population mobility and regional development so as to further improve the overall HDI of China in the future. To be specific, the government can make some efforts from the following aspects. First, the government should be devoted to removing obstacles for cross-regional population mobility. On the one hand, the household registration system should be promoted to help labor, particularly low-skilled labor, flow to large cities or urban agglomerations with high population densities and a high share of service industry. On the other hand, it is necessary to support cross-regional economic cooperation and provide relatively equal public services across regions (at least within urban agglomerations) for those who work in industries characterized by “technology diffusion” and strong externality.

Second, the government is supposed to adjust the direction and structure of public expenditure. In terms of the expenditure direction, it is important to reduce the direct investment of local government in industries and gradually transfer local finance into public finance. Particularly for those less developed regions, local expenditure should be oriented to infrastructure and education instead of productive investments. In terms of expenditure structure, the central government should constantly increase the transfer payment to those areas with net population outflow. It should be noted that the GDP per capita in the outflow areas will decline in a short time as the local government withdraws from the industrial investment. At the same time, the labor force outflow will also lead to the loss of tax revenues and reduce the future supply of public services in the long run, thus weakening the function of education and health in enhancing the HDI.

As for most developing countries, the improvement of HDI (at the welfare level) is realized in the process of urbanization and marketization, and the essence is the promotion of spatial allocation efficiency of production factors. In current China, one the one hand, it is a general trend for the population to flow and cluster in developed regions with the advancement of urbanization; on the other hand, the market-oriented reform will further promote the cross-regional allocation of production factors. Obviously, the impact of spatial factors on HDI is particularly important, such as the population density and regional integration. The findings of in this paper again demonstrate that the welfare level of some regions may be misestimated if a static view without a spatial perspective is adopted to evaluate the HDI in China, which may lead to the formulation of some inappropriate policies. For example, the supply of public services dominated by local government does not consider the law of population mobility, which may limit the improvement of welfare level. Besides, there are still administrative barriers to the mobility of production factors, which may decrease the spatial allocation efficiency of factors.

Finally, the shortcomings of this paper are also evident. We only incorporate the population density and regional integration into the HDI measurement, but there is a lack of deeper discussion on the mechanism underlying their effects on HDI. Besides, if smaller-scale panel data (at the municipal level, for example) are obtained and used, the impact of spatial factors on China’s HDI might be more comprehensively revealed. For example, for some provinces with a net outflow of population, they may have a net inflow of population at the level of cities or urban agglomerations if they are located in some important economic belts. Therefore, it is quite difficult to accurately capture the impact of population mobility on welfare at the provincial level. Surely, it is expected that the research of this paper can make the academic community and the government aware of the importance of spatial factors in the measurement of HDI and inspire more follow-up studies.

## 6. Conclusions

In the process of urbanization and marketization in China, the efficiency of cross-regional allocation of factors has significant impacts on the HDI, and ignoring spatial factors such as population density and regional integration may largely reduce the credibility of the HDI measurement. In this paper, an HDI with spatial factors is constructed with the spatial hierarchical factor model within the framework of Sen Capability Approach based on the provincial panel data in China from 2000 to 2018. The results reveal that: (1) provinces with high population densities and regional integration tend to have higher HDI rankings and low uncertainty, which can be attributed to the increase in education weights; (2) there is a spatial spillover effect in HDI with the intensification of spatial correlation year by year; and (3) there is a positive correlation between the spatial association and HDI ranking as indicated by the robust test with nighttime lighting as a proxy for GDP.

It is a common belief that population outflow and slowing of economic growth will lead to a prominent decline in regional welfare. However, the measurement of HDI in this paper indicates that it may not be entirely true. Even for large provinces with net population outflow (such as Henan and Sichuan), a higher degree of regional integration, such as being located in some important economic zones or urban agglomerations, can offset the negative effects of population outflow and thus achieve a more stable HDI ranking. Besides, the contribution of economic growth to welfare is not as significant as expected. In the HDI measured with the spatial hierarchical factor model, the weight of income shows some increases. However, these increases cannot be only attributed to economic growth, and are to some extent associated with the population density and urbanization rate as well. Further, we consider the spatial HDI as an alternative to the official HDI by incorporating factors such as population density and regional integration to discuss the important influence of spatial factors on the welfare level of each region in China. This paper does not essentially change the ranking of traditional HDI. The core purpose is to illustrate that the importance of employment clustering and urban agglomeration to the promotion of welfare will be largely ignored if spatial factors such as population density and regional integration are not considered in a country as large as China, which may violate the observation of “mobility brings prosperity” in reality and cause theoretical difficulty in explaining the heterogeneity of regional welfare, as well result in the formulation of some policies against the laws of population flow and economic growth.

## Figures and Tables

**Table 1:** Top 10 Provinces with the Largest Difference Between Traditional HDI and Spatial HDI Rankings.

Province	Ranking		Difference
	Traditional HDI	Spatial HDI	
Heilongjiang	18	12 (11, 12)	6
Chongqing	13	19 (17, 20)	6
Jilin	15	10 (9, 10)	5
Hebei	20	15 (14, 16)	5
Hainan	10	15 (14, 16)	5
Shanxi	22	17 (13, 21)	5
Ningxia	25	21 (19, 22)	4
Hubei	11	14 (13, 15)	3
Hunan	17	20 (18, 22)	3
Anhui	23	26 (25, 27)	3

Note: In traditional HDI, we use the equal weighting method, that is, the same weight is assigned to each of the three secondary indicators of health index, education index and income index. In spatial HDI, by referring to the normalizing “squared correlation coefficient” method of Hogan and Tchernis [25], the weight for the health index is calculated to be 0.3255, that for the education index is 0.3544 and that for the income index is 0.3201.

**Table 2:** Estimation Results of the Spatial Spillover Effect of HDI.

Explained Variable: HDI		
	Model 1: FE-SAR	Model 2: GMM-SAR
Spatial spillover of HDI (*ρ*)	0.124 *** (0.032)	0.064 ** (0.030)
Undersufficient Identification LM Test		311.272 ***
Overidentification Hansen J Test		Pass
Observed Value	589	589

Note: (1) ** significantly different at the 5 percent significance level and *** 1 percent significance level; (2) SE in bracket; (3) maximum likelihood estimation (MLE) is applied to both model 1 and model 2.

**Table 3:** Cross Section Estimation Results of the Spatial Spillover Effect of HDI.

Explained Variable: HDI			
	Model 3: FE-SAR		Model 4: FE-SAR
Spatial Spillover in 2001 (*ρ*)	0.018 *** (0.003)	Spatial Spillover in 2010 (*ρ*)	0.114 *** (0.005)
Spatial Spillover in 2002 (*ρ*)	0.031 *** (0.003)	Spatial Spillover in 2011 (*ρ*)	0.137 *** (0.006)
Spatial Spillover in 2003 (*ρ*)	0.044 *** (0.003)	Spatial Spillover in 2012 (*ρ*)	0.144 *** (0.006)
Spatial Spillover in 2004 (*ρ*)	0.055 *** (0.003)	Spatial Spillover in 2013 (*ρ*)	0.153 *** (0.006)
Spatial Spillover in 2005 (*ρ*)	0.060 *** (0.003)	Spatial Spillover in 2014 (*ρ*)	0.156 *** (0.006)
Spatial Spillover in 2006 (*ρ*)	0.077 *** (0.004)	Spatial Spillover in 2015 (*ρ*)	0.162 *** (0.006)
Spatial Spillover in 2007 (*ρ*)	0.091 *** (0.004)	Spatial Spillover in 2016 (*ρ*)	0.165 *** (0.007)
Spatial Spillover in 2008 (*ρ*)	0.102 *** (0.005)	Spatial Spillover in 2017 (*ρ*)	0.172 *** (0.007)
Spatial Spillover in 2009 (*ρ*)	0.113 *** (0.005)	Spatial Spillover in 2018 (*ρ*)	0.176 *** (0.007)

Note: Note: (1) *** significantly different at the 1 percent significance level; (2) The estimation results for the first year (2000) are missed due to the use of FE-SAR estimation.

## Data Availability

The data presented in this study are available on request from the corresponding author.
